# A broadband reflective-type half-wave plate employing optical feedbacks

**DOI:** 10.1038/s41598-017-09561-y

**Published:** 2017-08-22

**Authors:** Gaochao Zhou, Bo Zhu, Junming Zhao, Guanghao Zhu, Biaobing Jin, Yijun Feng, Lin Kang, Weiwei Xu, Jian Chen, Peiheng Wu

**Affiliations:** 1 School of Electronic Science and Engineering, Nanjing University, Nanjing, 210023 China; 2 Cooperative Innovation Centre of Terahertz Science, University of Electronic Science and Technology, Chengdu, China

## Abstract

We propose and demonstrate a type of a broadband half-wave plate that operates in the reflective mode. It consists of a metal grating embedded in a dielectric slab and placed on top of a grounded metal surface. We theoretically show that owing to the optical feedback effect which originates from the wave reflections at the air-dielectric interface, the proposed half-wave plate exhibits a broadened and flattened response when comparing to the case where the feedback effect is absent. Such a prediction is validated using both numerical and experimental works carried out on a half-wave plate designed at 10 GHz. Moreover, our theoretical analysis also reveals that the half-wave plate has an interesting feature of broad angular response. Taking advantage of these features, we experimentally demonstrate that the proposed device can function as a freely tunable linear polarization converter with polarization conversion residues less than *−*20 dB in a wide frequency band, under the condition that the incident angle is as large as 45 degrees.

## Introduction

Metasurface^1, 2^ refers to a general class of planar structures that consist of subwavelength scale elements called as the unit cells. It has a close analogy to and also represents a much enriched extension of the frequency selective surface originally proposed for microwave electronics^3^. One of the most fascinating features of the metasurface is its electromagnetic response that can be engineered almost at will. Taking advantage of this feature, a variety of intriguing wave phenomenon such as the generalized Snell law for reflection and refraction^4^ and the high resolution light focusing for plane waves^5^ have been demonstrated using ultrathin optical structures.

Some other important engineering outcomes enabled by the development of the metasurface may include the polarization manipulators^6–15^, and in particular the linear polarization converters. For linear polarization converters, it is well known that they can be separated into the transmitting cases^16–21^ and the reflecting cases^22–33^. Comparing to the transmitting cases, linear polarization converter working in the reflecting cases has a unique advantage of nearly 100% device efficiency, since here by definition the problem of impedance matching is absent. Note that viewed from a practical perspective, the development of the reflective type linear polarization converter encounters two demanding issues that need to be carefully addressed. The first issue is the spectral bandwidth issue, which can be solved, e.g., using the technique of polarization selective resonant structure backed by a grounded plane^24–33^. The second issue is the incidence angle issue since in order to conveniently separate the output beam from the input beam, the half-wave plate should be capable to accept incident angles as large as a few tens of degrees. We emphasize that in general, these two issues of the reflective type half-wave plate may not be resolved simultaneously.

In this article, we propose and demonstrate a kind of reflective type linear polarization converter (half-wave plate) that can possess broad spectral and angular bandwidths at the same time. We start our device design by first considering a simple version of the reflective type half-wave plate that consists of a metal grating placed on top of a grounded metal surface. We next show that the spectral bandwidth of the half-wave plate can be broadened by enclosing the metal grating with a dielectric slab, which introduces a beneficial optical feedback effect due to the wave reflections occurring at the air-dielectric interface. Moreover, it is also analytically proven that the proposed device has a feature of broad angular bandwidth. Based on these features, we propose and demonstrate that the conceived device can function as a broadband freely tunable linear polarization rotator that can work with an incident angle as large as 45 degrees.

## Result

### Design principle

**Figure 1:**
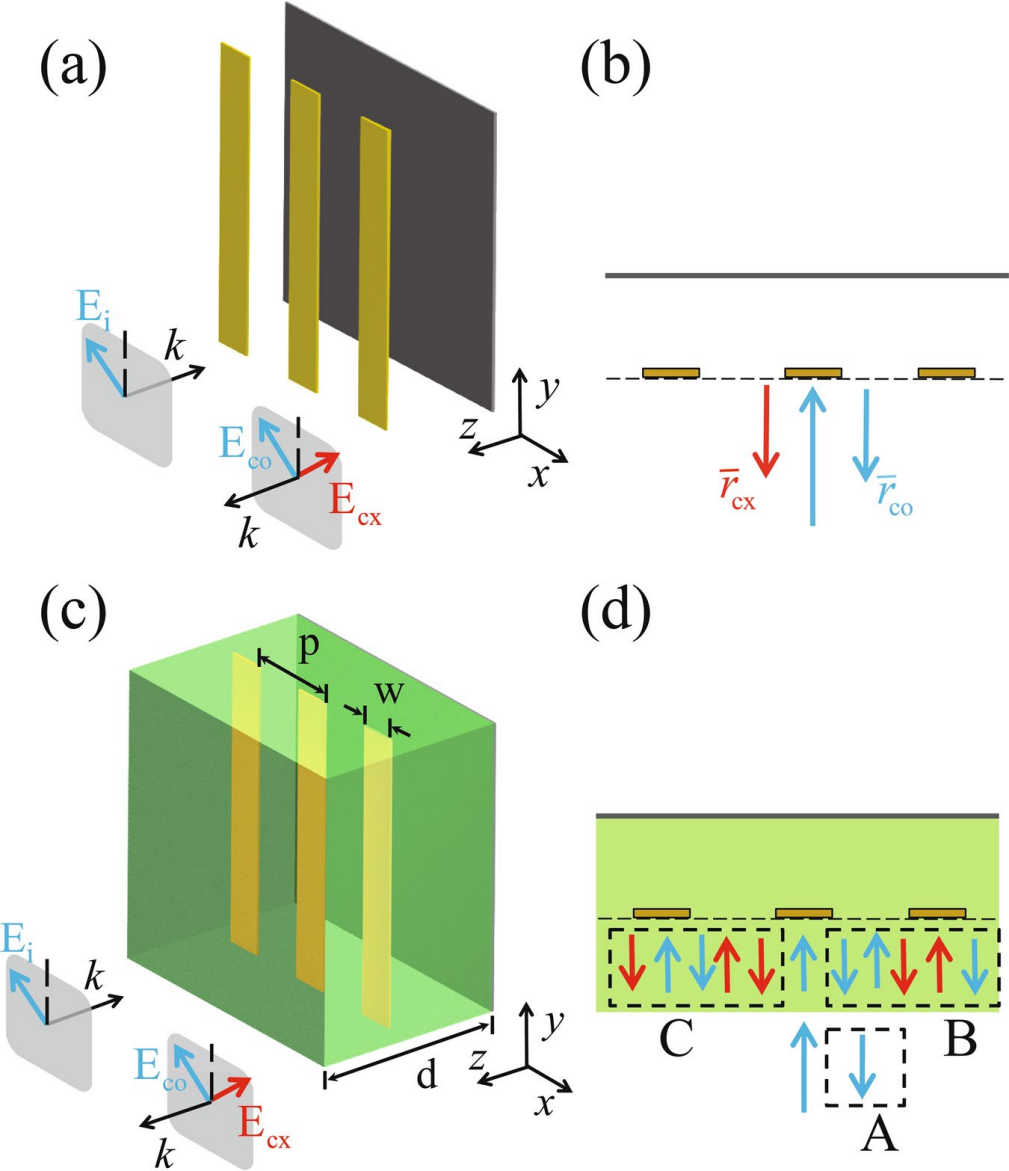
Illustrations on the design and operating principle of the proposed device. (**a**) Schematics of a half-wave plate with a narrow bandwidth. (**b**) Analytic model for treating the half-wave plate depicted in (**a**). (**c**) Schematics of the proposed half-wave plate with a broad spectral bandwidth. (**d**) Analytic model for treating the half-wave plate depicted in (**c**). Note that the blue and red colors are in correspondence to cases where the polarization angles are *−*45 degrees and 45 degrees with respect to the grating wires.

Figure 1(a) sketches the start point of our device design, which is a simple version of the half-wave plate that operates in the reflective mode. It consists of a metal grating and a grounded metal surface. When the pitch and duty cycle of the metal grating are properly arranged, the metal grating allows waves with polarization perpendicular to the grating wires to be efficiently transmitted, while makes waves with polarization parallel to the grating wires to be efficiently reflected. In other words, the metal grating and the grounded metal surface can be viewed as virtual reflectors for cases of parallel and perpendicular polarizations respectively. By setting the separation between the metal grating and the grounded metal surface to be a quarter of the wavelength of interest, the phase difference of the reflected waves between cases of perpendicular and parallel polarizations will differ by an amount of π, rendering the structure to function as a half-wave plate. We note that because the polarization related phase difference is determined by the separation between the two virtual reflectors, which is a fixed value upon the completion of the device fabrication, the bandwidth of the half-wave plate depicted by Fig. 1(a) would be narrow.

To increase the bandwidth, consider a situation where the normally incident wave has a polarization aligned along the direction *−*45 degrees to the grating wires, as illustrated by Fig. 1(a). Note that at the desired wavelength of interest where the quarter-wavelength condition is strictly satisfied, the reflected wave would have a polarization exactly aligned along the direction 45 degrees to the grating wires (cross-polarized) due to the perfect polarization conversion effect of the ideal half-wave plate. However, when the operating wavelength deviates from the desired one and the quarter-wavelength condition breaks down, the polarization conversion process will be incomplete and the reflected beam will contain some component of which the polarizations is *−*45 degrees oriented with respect to the grating wires (co-polarized). Referring to Fig. 1(b), in the vicinity of the desired wavelength and at the level of first order accuracy, the amplitude coefficients for the cross-polarized and co-polarized components (denoted by ${\overline{r}}_{{\rm{cx}}}$ and ${\overline{r}}_{{\rm{co}}}$ respectively) of the reflected beam are given by

1 $${\overline{r}}_{{\rm{cx}}}=\frac{{e}^{i2(\frac{\pi }{2}+{\rm{\Delta }}\alpha )}-1}{2}\approx -1-i{\rm{\Delta }}\alpha $$

and

2 $${\overline{r}}_{{\rm{co}}}=\frac{-{e}^{i2(\frac{\pi }{2}+{\rm{\Delta }}\alpha )}-1}{2}\approx i{\rm{\Delta }}\alpha $$

respectively, where ∆α denotes the shift of the propagation phase (defined between the metal grating and the grounded metal surface) apart from π/2, as caused by the working wavelength detuning. We emphasize that the existence of the co-polarized component of the reflected beam, i.e. r_co_, is a direct indication that the performance of the half-wave plate is deteriorated. By eliminating it with the help of certain means, the bandwidth of the half-wave plate will be broadened.

Based on the above arguments, we propose an optical feedback method to eliminate the co-polarized component of the reflected beam and consequently increase the bandwidth of the half-wave plate. Here the idea is to feed the co-polarized component of the reflected beam back to the grounded-grating structure and reutilize the polarization conversion effect of such a structure for suppressions. Figure 1(c) sketches the proposed device that is designed according to such an idea. It consists of a metal grating embedded inside a dielectric slab and placed on top of a grounded metal surface. Note that our theoretical analysis presented in the Methods section indicates that for optimized performance the metal grating should be positioned at the middle plane of the dielectric slab, of which the thickness is half of the wavelength of interest. Comparing to the case of Fig. 1(a), it can be seen that here in the case of Fig. 1(c) a dielectric slab is added. The addition of the dielectric slab creates an air-dielectric interface, which introduces an optical feedback effect by reflecting the co-polarized component back to the device kernel, i.e. the grounded-grating structure. The co-polarized component of the reflected beam then undergoes further rounds of polarization conversion processes and in an intuitive sense, may be cancelled out with appropriate arrangements.

We unitize a multiple-reflection model to determine the optimal condition for the cancellation of the co-polarized component of the reflected beam. As being illustrated by Fig. 1(c) and (d), when the incident wave is normally launched with a polarization aligned along the direction *−*45 degrees to the grating wires, there exists three channels where the co-polarized components can be generated. The first channel is the direct reflection channel since when the incident wave impinges onto the air-dielectric interface, it will be partially reflected due to the mismatch of the air and dielectric impedances. Such a channel is denoted by letter A in Fig. 1(d). The reflected wave produced in this channel is completely co-polarized and has an amplitude coefficient of

3 $${r}_{{\rm{A}}}=r,$$

where r denotes the reflection efficiency of the air-dielectric interface for the case of air side incidence. Upon the reflection, the incident wave will continue to propagate into the dielectric slab and be reflected back by the grounded-grating structure. The reflected beam from the grounded-grating structure has two components, with one being co-polarized and the other being cross-polarized. These two polarization components will subsequently undergo multiple reflections between the air-dielectric interface and the grounded-grating structure, and create the second and third channels for the co-polarized components. Such two channels are denoted by letters B and C in Fig. 1(d). We remark that for the multiple-reflection processes, reflections from the grounded-grating structure will alter the polarization state while reflections from the air-dielectric interface will preserve the polarization state. It therefore takes four reflections for the trapped beam to complete a cycle, as being illustrated by the five arrows drawn with different colors in the dashed box B and C of Fig. 1(d). The two channels of B and C are coupled to the external space through wave transmissions at the air-dielectric interface. With some manipulations, the amplitude coefficients of the co-polarized components from these two channels read as

4 $${r}_{{\rm{B}}}=\frac{t\cdot {\overline{r}}_{{\rm{co}}}\cdot t^{\prime} \cdot {e}^{i2(\frac{\pi }{2}+{\rm{\Delta }}\alpha )}}{1-q}$$

and

5 $${r}_{{\rm{C}}}=\frac{t\cdot {\overline{r}}_{{\rm{cx}}}\cdot r^{\prime} \cdot {\overline{r}}_{{\rm{cx}}}\cdot t^{\prime} \cdot {e}^{i4(\frac{\pi }{2}+{\rm{\Delta }}\alpha )}}{1-q},$$

where t denotes the transmission efficiency of the air-dielectric interface for the case of air side incidence, r’ and t’ denote the reflection and transmission efficiencies of the air-dielectric interface for the case of dielectric side incidence, ${\overline{r}}_{{\rm{cx}}}$ and ${\overline{r}}_{{\rm{co}}}$ are given by Eqs (1) and (2) respectively, and

6 $$q=r^{\prime} \cdot {\overline{r}}_{{\rm{cx}}}\cdot r^{\prime} \cdot {\overline{r}}_{{\rm{cx}}}\cdot {e}^{i4(\frac{\pi }{2}+{\rm{\Delta }}\alpha )}$$

denotes the cycle gain of the multiple reflection processes as experienced by the trapped beam. Summing up contributions from these three channels, the overall amplitude coefficient for the co-polarized component of the reflected beam reads as

7 $${r}_{{\rm{co}}}\approx \frac{{r}^{2}-6r-1}{{r}^{2}-1}i{\rm{\Delta }}\alpha .$$

Equation (7) indicates that in order to cancel the co-polarized component of the reflected beam at the level of first order accuracy, r needs to be *−*0.16 or equivalently speaking the dielectric slab needs to have a permittivity of 1.92. Note that in deriving the above result, higher order polarization conversion processes have been neglected. If these processes are taken into considerations, it can be shown that the exact value of the permittivity of the dielectric slab is 2 and the cancellation is at the level of second order accuracy. Please refer to the Methods section for a rigorous proof.

Before we proceed, we remark that the proposed broadband half-wave plate should be distinguished from those that are constructed solely using the metasurfaces, since here multiple λ/4 dielectric slabs are involved. Several other related broadband devices that also employ multiple λ/4 dielectric slabs include the one proposed by Grady *et al*.^6^ and very recently the one proposed by Pisano *et al*.^18^. The much increased bandwidths for these devices are attributed to the multiple reflections occurring at the interfaces of the dielectrics. Note that a similar effect could also be found in broadband impedance matching networks that employ multiple λ/4 transmission line segments^34^.

### Numerical and experimental validations

We use a reflective type half-wave plate designed at 10 GHz to validate the above analysis. Referring to Fig. 1(c), the wire grating part of the designed device has a pitch of p = 2 mm and a width of w = 0.8 mm. It is confirmed numerically that this arrangement allows the wire grating to be highly transparent for incident waves with perpendicular polarizations but highly reflective for incident waves with parallel polarizations. The dielectric slab part of the designed device has a thickness of d = 10 mm. As a result, the conceived device would have a central working frequency near 10 GHz when the dielectric slab has a permittivity close to 2.

**Figure 2:**
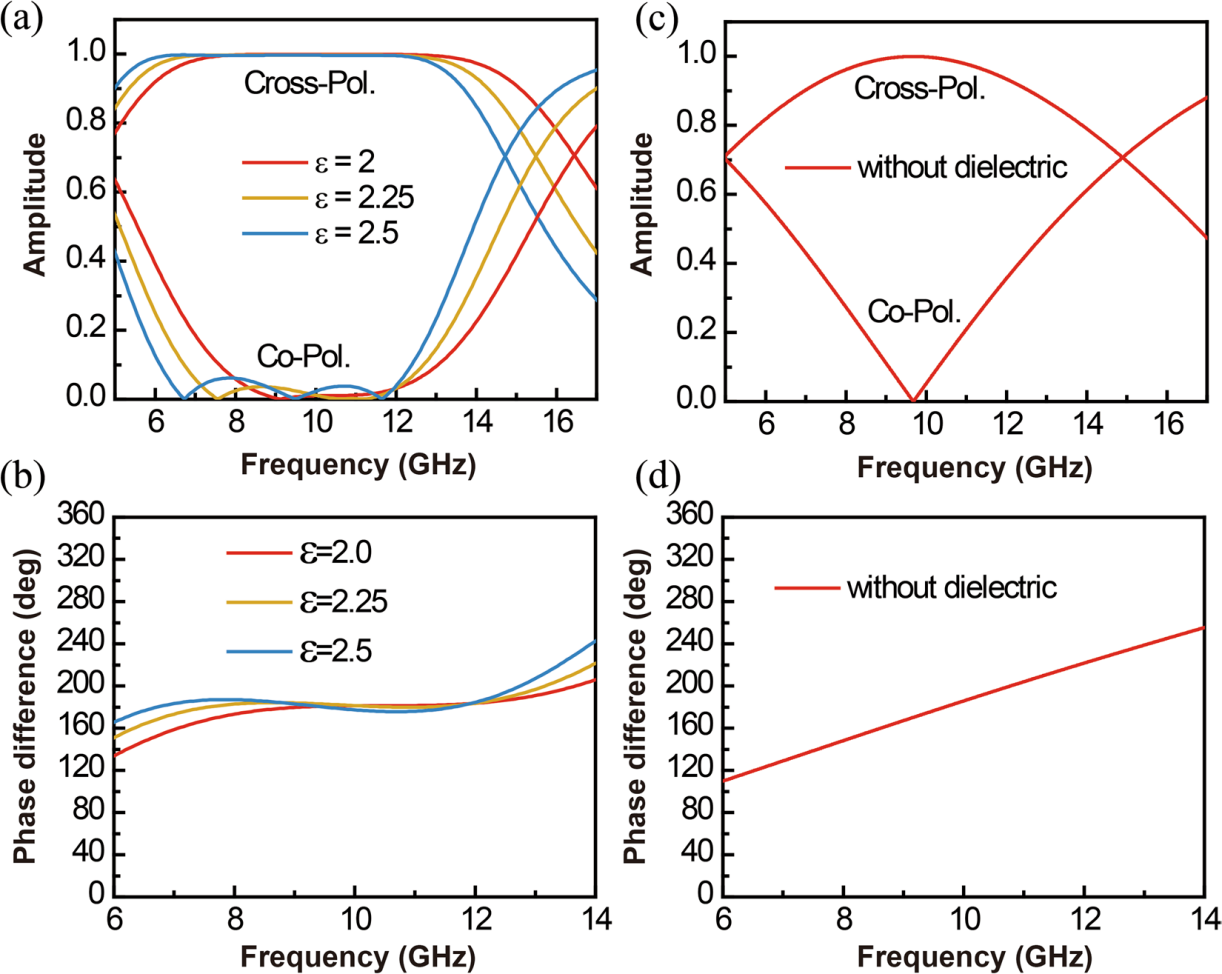
Numerical results for the proposed half-wave plate under normal incidence. (**a**) Amplitude coefficients for the co-polarized and cross-polarized components of the reflected beam. (**b**) Phase difference of the reflected beams between cases where the polarizations of the input beam are parallel and perpendicular to the grating wires. For comparison purpose, (**c**) and (**d**) sketch the response curves obtained for the narrowband design case where the dielectric slabs is absent.

Figure 2(a) and (b) summarize the numerical results, i.e. the amplitude coefficients for the co-polarized and cross-polarized components and the polarization related phase difference, for the designed broadband device under the condition of normal incidence. Note that in our simulations (carried out with the CST microwave studio), we have assumed that the metal has a conductivity and a thickness of 5.8 × 10^7^ S/m and 0.035 mm respectively, and the dielectric has a loss tangent of 0.001. Three different cases where the dielectric slabs have permittivities of 2, 2.25 and 2.5 are considered, in correspondence to the curves drawn with red, yellow and blue colors respectively. In comparison, Fig. 2(c) and (d) summarize the response curves for the narrowband design case, i.e. the case where the dielectric slab is absent. Comparing Fig. 2(a) and (b) with 2(c) and (d), it can be readily identified that with the addition of the dielectric slab, the spectral responses are significantly broadened and flattened. We remark that Fig. 2(a) and (b) also reveal that the proposed half-wave plate works best in term of the band flatness when the dielectric slab has a permittivity of 2. This is in an agreement with our theoretical prediction presented in the Methods section. When the permittivity of the dielectric slab varies from 2 to 2.5, although ripples appear in the response curves, however, the amplitude coefficient of the co-polarized component of the reflected beam remains less than 0.1 (*−*20 dB for intensity) in a broad frequency band. The good tolerance on the dielectric permittivity of the proposed device greatly alleviates the restrictions for the selection of the slab materials.

**Figure 3:**
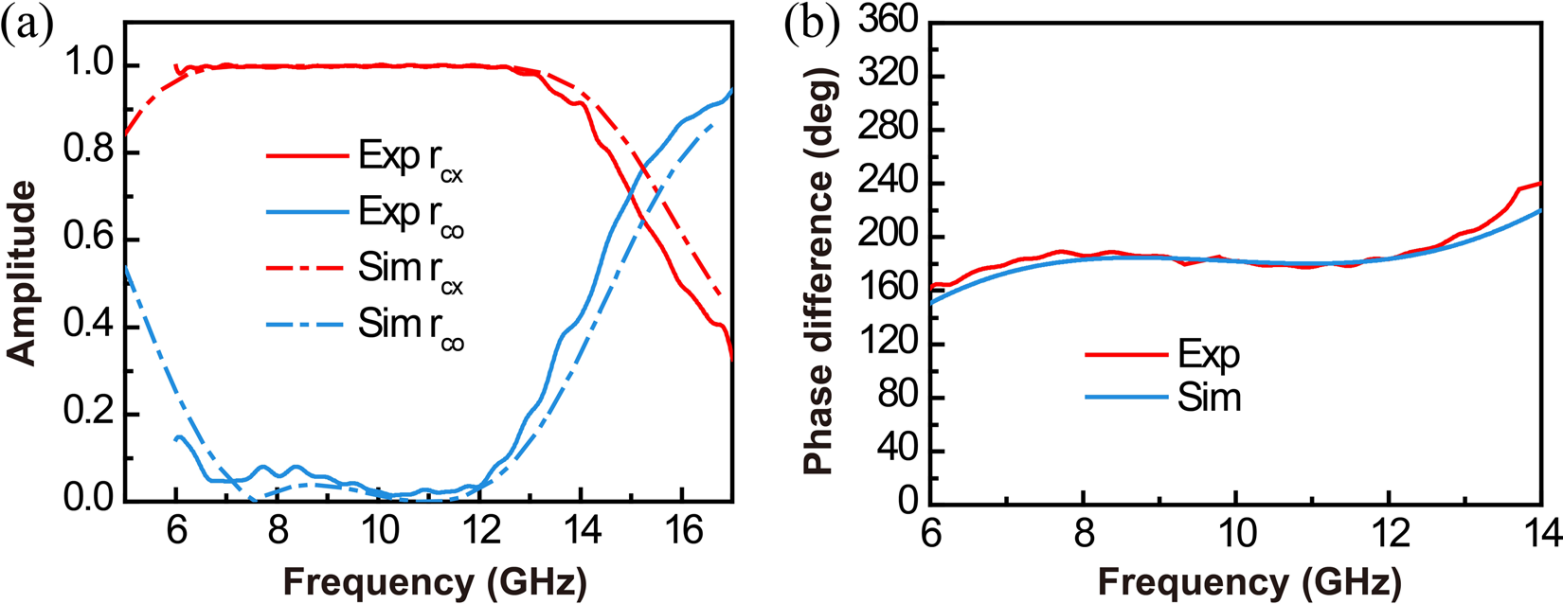
Experimental and numerical results for the proposed half-wave plate operating under quasi-normal incidence. (**a**) Amplitude coefficients for the co-polarized and cross-polarized components of the reflected beam. (**b**) Phase difference of the reflected beams between cases where the polarizations of the input beam are parallel and perpendicular to the grating wires. Note that the incident angle is 5 degrees.

To provide further validations, samples of the half-wave plate with size of 32 cm by 32 cm are fabricated and tested. Note that in fabricating the tested device, we first print a metallic grating onto a grounded dielectric slab, and then attach a second dielectric slab with the same thickness on top of it. We use copper for the metal and F4BK for the dielectric. The copper structure has a thickness of 0.035 mm and F4BK has a permittivity of 2.25. Such a permittivity is expected to lead to a good device performance according to the simulation results obtained previously. The fabricated samples are mounted onto an absorbing screen and are tested using a microwave network analyzer connected to a transmitting antenna and a receiving antenna (both are placed vertically). The transmitting and receiving angles of the two antennas are set to be ±5 degrees with respect to the normal direction of the half-wave plate. This allows normal incidence condition to be approximately achieved in the measurements. By arranging the fabricated device in manners where the grating wires are vertically oriented and horizontally oriented, two sets of data for the reflection phases are obtained. Their difference is plotted by the red curve shown in Fig. 3(b), where it can be seen that the phase difference has a flat plateau pinned at 180 degrees in the vicinity of 10 GHz. The measured result is in a good agreement with the numerical one shown by the blue curve. Moreover, by manipulating the measurement data, the amplitude coefficients for the co-polarized and cross-polarized components of the reflected beam are also obtained for the case where the incident beam is *−*45 degrees polarized. The results are sketched by the solid curves in Fig. 3(a), in together with the numerical ones drawn by the dashed curves. The good agreement between the experimental and numerical data shown in Fig. 3 confirms that the proposed device has a broadened operating bandwidth by effectively suppressing the co-polarized component of the reflected beam.

**Figure 4:**
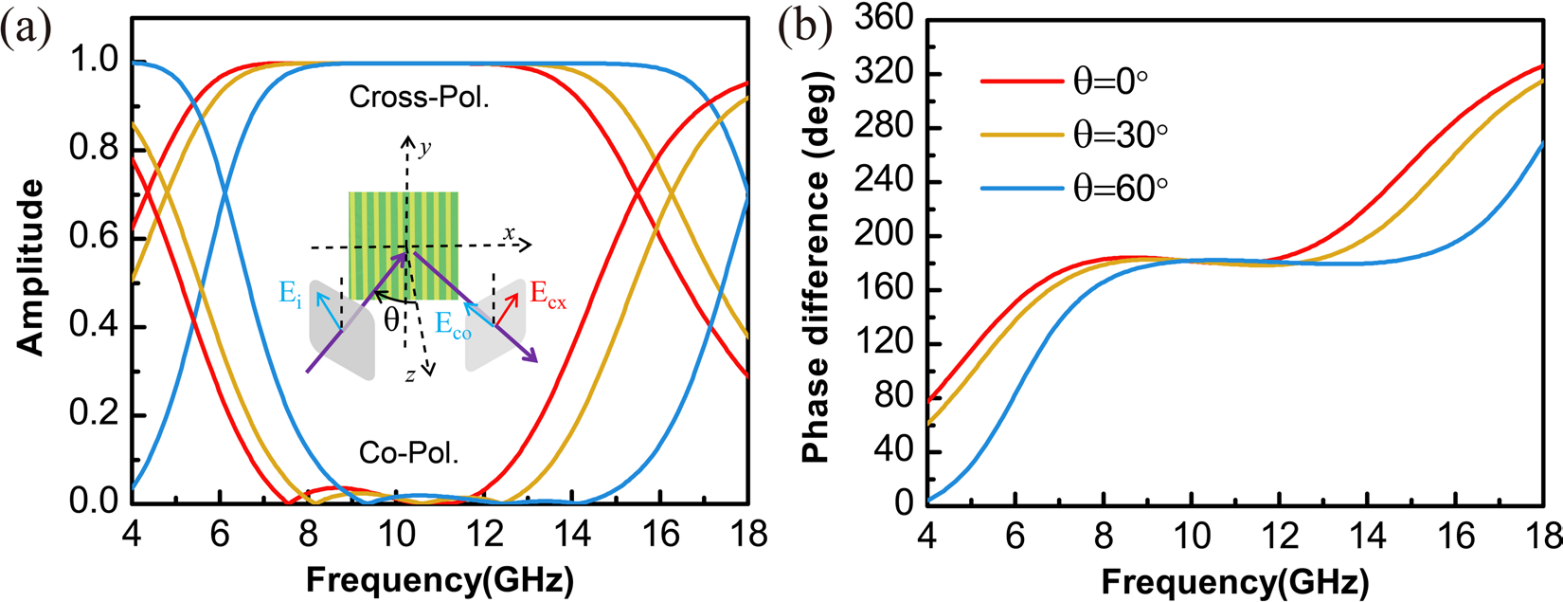
Numerical results for the proposed half-wave plate under oblique incidence. (**a**) Amplitude coefficients for the co-polarized and cross-polarized components of the reflected beam. (**b**) Phase difference of the reflected beams between cases where the polarizations of the input beam are parallel and perpendicular to the grating wires. The insect in (**a**) shows the oblique incidence situation under consideration.

We emphasize that in order to make the proposed device practically useful, in addition to the requirement of the broad spectral bandwidth, it is also preferred that the device can work with incident angles as large as a few tens of degrees, since larger incident angles provide greater convenience for the experiments. It is thus necessary to study the device performance under cases of oblique incidences. To this end, referring to the inset of Fig. 4(a), we tilt the incidence wave-vector (also the input plane marked by grey) by an angle of θ in the x-z plane, while keep the input polarization state relatively unchanged. Consequently, the wave-vector of the reflected beam (also the output plane marked by grey) will be tilted by an angle of −θ in the x-z plane, and the definitions of the co-polarized and cross-polarized components of the reflected beam remain relatively unchanged. With such an arrangement being emulated in the numerical simulations (the permittivity of the dielectric slab is 2.25), the amplitude coefficients of the co-polarized and cross-polarized components for the reflected beam are calculated for three cases where the incident angles θ are 0, 30 and 60 degrees. The obtained results are sketched by the curves drawn with red, yellow and blue colors in Fig. 4(a) respectively. It can be seen that other than a shift of the central working frequency, the performance of the half-wave plate does not suffer any deterioration even if the incident angle is as large as 60 degrees. Moreover, we have also calculated the phase difference of the reflected beams between cases where the polarizations of the input beams are parallel and perpendicular to the grating wires under oblique incidences. The results are presented in Fig. 4(b), which are in support of Fig. 4(a). It is hence numerically demonstrated that the proposed half-wave plate is insensitive to the incident angles except a shift of the central working frequency. Such a numerical result is in agreement with the theoretical prediction drawn in the Methods section.

**Figure 5:**
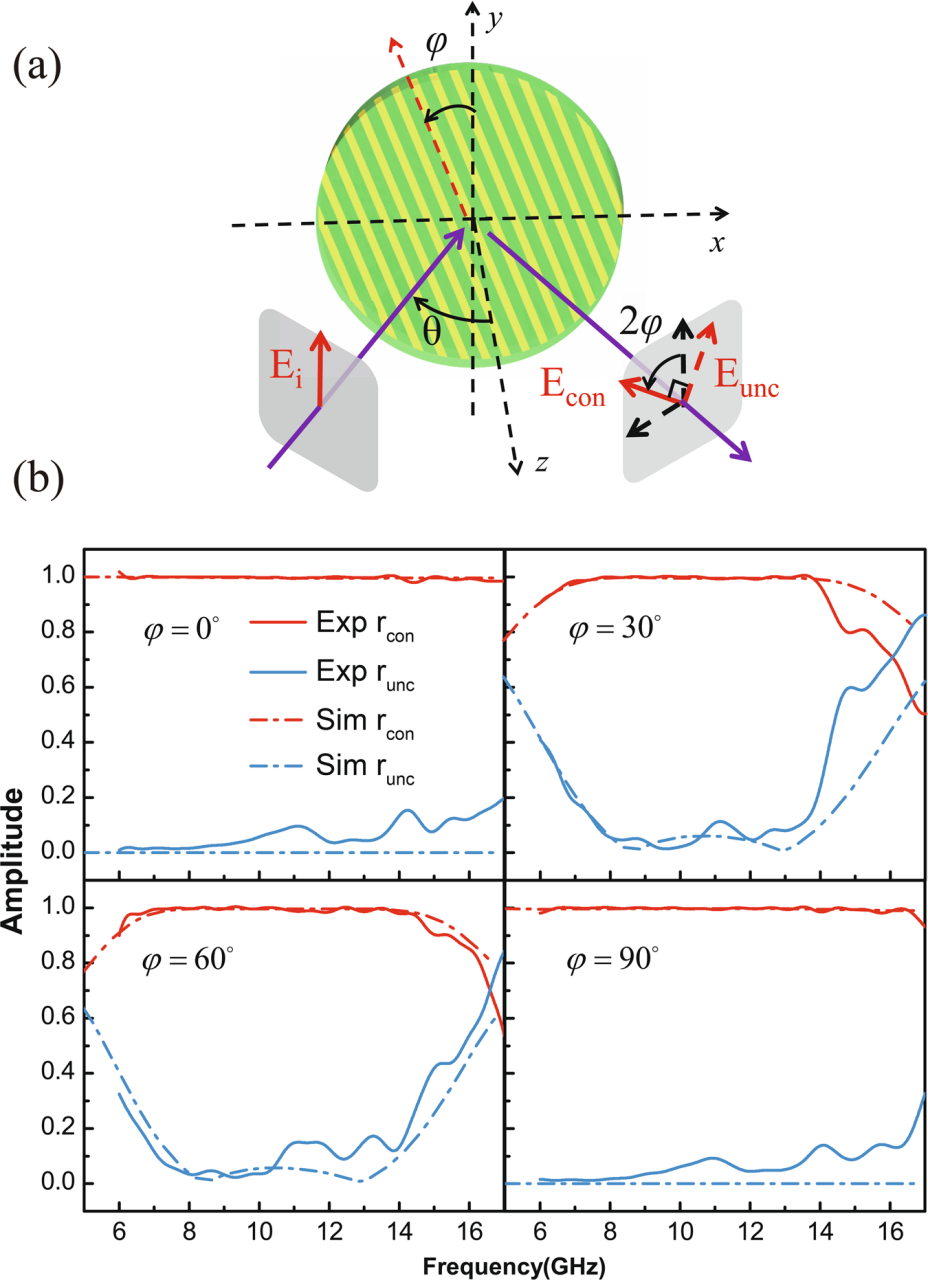
Application of the proposed half-wave plate as a freely tunable linear polarization converter. (**a**) Illustration on the device operation. The input beam has a fixed polarization along the y direction. When the half-wave plate is rotated by an angle of *φ*, the polarization of the reflected beam will be rotated by an angle of 2*φ*. (**b**) Experimental and numerical results for the tunable polarization converter for cases where the half-wave plate is rotated by 0, 30, 60 and 90 degrees and the incident angle is 45 degrees. Note that *r*_con_ and *r*_unc_ denote the amplitude conversion efficiencies from the polarization state marked by *E*_i_ into the polarization states marked by *E*_con_ and *E*_unc_ respectively.

Motivated by the broad spectral and angular responses of the conceived half-wave plate, we propose to function it as a polarization converter that can rotate the linear polarization of the output beam by arbitrary angles. Figure 5(a) sketches the details of the proposed operation. The incident beam has an input angle of θ in the x-z plane and is vertically polarized along the y axis (denoted as E_i_). The half-wave plate is placed in the x-y plane with the grating wires being rotated by an angle of φ away from the y direction. Due to the polarization rotation effect of the half-wave plate, in an ideal situation, the polarization of the reflected beam would be rotated by an angle of 2φ in the output plane (referred as the polarization converted beam and denoted as E_con_). The non-ideal operation of the half-wave plate will yield some reflection components being perpendicular to the polarization converted beam (referred as the polarization unconverted beam and denoted as E_unc_). Note that since φ can be arbitrarily adjusted by rotating the half-wave plate, the linearly polarized state of the reflected beam thus can be freely tuned. To evaluate how much portion of E_i_ is converted to E_con_ and how much portion of E_i_ is left as E_unc_, experimental measurements are carried out. In performing the experiments, the incident angle θ is fixed at 45 degrees and the input beam is fixed as y-polarized. The grating angle φ has four different values to be tested, i.e., 0, 30, 60 and 90 degrees. Note that for each selected grating angle of φ, the amplitude coefficients of the polarization converted and unconverted components (denoted by *r*_con_ and *r*_unc_ respectively) can be determined by the formulas that read as

8 $${r}_{\text{con}}={r}_{{\rm{h}}}\,\cos \,\,2\phi +{r}_{{\rm{v}}}\,\sin \,\,2\phi $$

and

9 $${r}_{{\rm{unc}}}={r}_{{\rm{h}}}\,\sin \,\,2\phi -{r}_{{\rm{v}}}\,\cos \,\,2\phi ,$$

where r_h_ and r_v_ denote the measured reflection coefficients for cases where the transmitting and receiving antennas are both horizontally polarized and are both vertically polarized respectively. By measuring r_h_ and r_v_ respectively and with the help of Eqs (8) and (9), the amplitude coefficients for the polarization converted and unconverted components are obtained. Their results are summarized in Fig. 5(b), in together with the numerically simulated ones. It can be seen that under the condition of 45 degrees incidence, the proposed device can freely rotate the linear polarization of the reflected beam, while leaving very low polarization unconverted residues in a broadband manner. The demonstrated performance of the proposed reflective type half-wave plate suggests that it might be of interest for various realistic applications.

We remark that in addition to the advantage of broad spectral and angular responses, our device also has other beneficial features such as easiness of fabrication and widely extendable frequency bands. For the first feature, we note that according to the design principle, the metal grating needs to be placed at the middle plane of the dielectric slab, as being analytically proven in the Methods section. In other words, the top and bottom half parts of the dielectric slab need to have the same thickness, which can be realized by growing them consecutively under the same fabrication condition. Moreover, due to the broad spectral response, there is no strict requirement on the absolute thickness of the dielectric slab. For the second feature, we note that by scaling down the dimension of the proposed device, its operating frequency band can be extended into the terahertz and optical ranges, where for example F4BK and SiO_2_ (ε~2) can be used as the dielectric materials respectively.

## Conclusion

In conclusion, we have proposed and demonstrated a type of broadband half-wave plate that operates in the reflective mode. It consists of a metal grating placed inside a dielectric slab and backed by a grounded metal surface. We show that the spectral response of the proposed device can be significantly broadened and flattened with the help of optical feedbacks, which originate from the reflections occurring at the air-dielectric interface. The proposed device also has a feature of broad angular response. Taking advantage of these beneficial features, it is experimentally demonstrated that the proposed device can function as a broadband freely tunable linear polarization converter, under the situation that the incident angle is as large as 45 degrees.

## Methods

**Figure 6:**
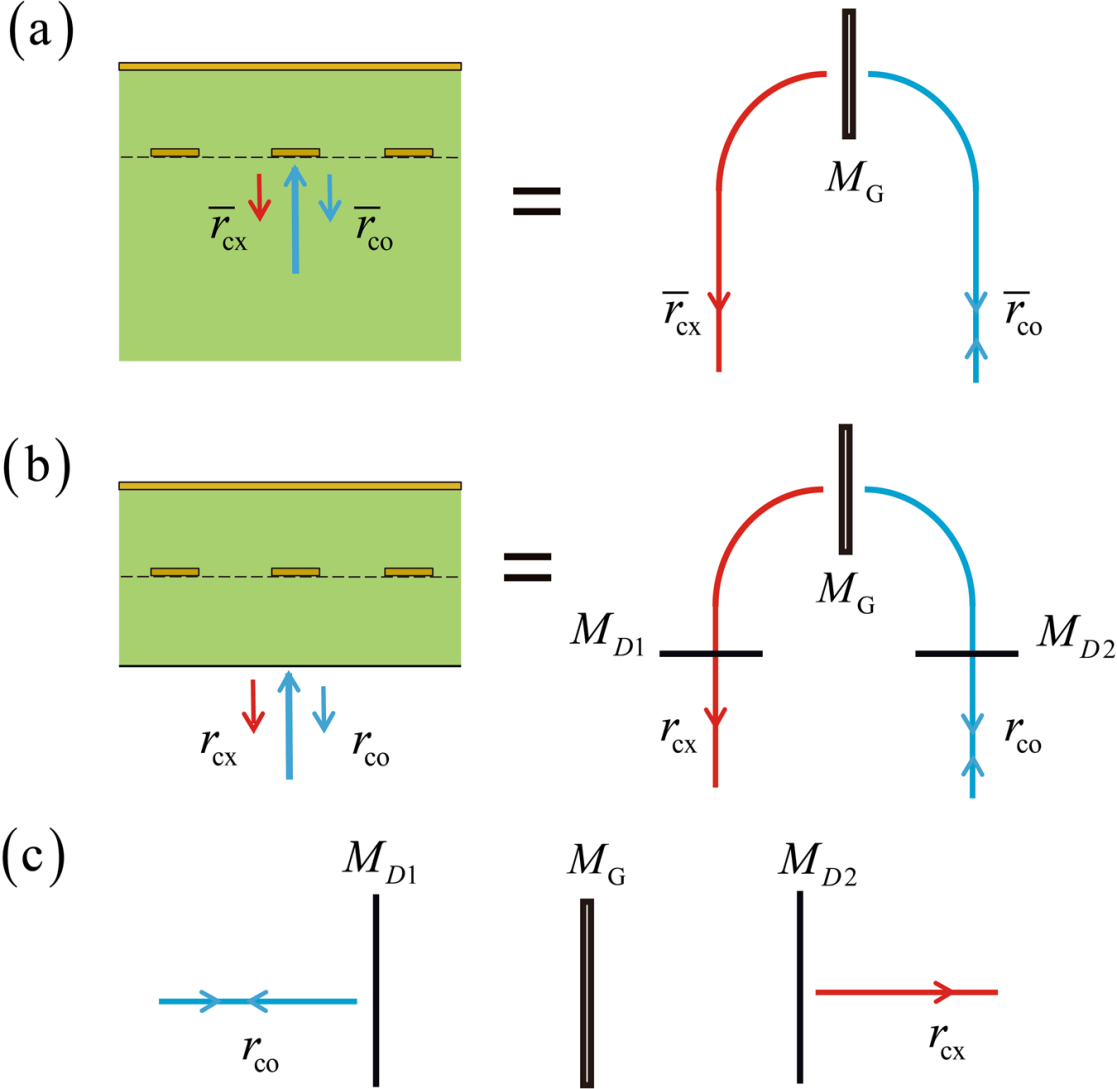
Signal-flow picture for the rigorous analysis of the proposed broadband half-wave plate. (**a**) Case where the grounded dielectric slab has an infinite thickness. (**b**) Case where the grounded dielectric slab has a finite thickness. (**c**) The deduced signal-flow chart for the rigorous analysis of the proposed broadband half-wave plate. Note that the blue and red colors denote the co-polarized and cross-polarized channels respectively.

We employ a signal-flow chart based method to analytically prove that the exact value of the optimized permittivity of the dielectric slab is equal to 2. Shown by the left part of Fig. 6(a), a metal grating is embedded inside a semi-infinite dielectric medium terminated by a grounded metal surface. Consider a situation where the incident wave is normally launched with a polarization aligned along the direction *−*45 degrees to the grating wire. Due to the polarization conversion effect of the grounded-grating structure, the reflected wave has two polarization components, with one being co-polarized (marked by the blue arrow and with a reflection efficiency denoted by ${\overline{r}}_{{\rm{co}}}$) and the other being cross-polarized (marked by the red arrow and with a reflection efficiency denoted by ${\overline{r}}_{{\rm{cx}}}$). The corresponding signal-flow chart is sketched by the right part of Fig. 6(a), where the signal-flow paths colored by blue and red denote the polarization channels which are co-polarized and cross-polarized respectively. The virtual mirror denoted by *M*_*G*_ is in correspondence to the grounded-grating structure. It plays the role of signal coupling between the blue and red channels. Figure 6(b) describes the situation where the semi-infinite dielectric medium reduces to a dielectric slab and the metal grating is place at the central plane of the dielectric slab. In this situation, due to the reflection effect at the dielectric-air interface, two virtual mirrors marked by *M*_D1_ and *M*_D2_ are introduced.

The signal-flow chart drawn by the right part of Fig. 6(b) can be transformed to the one drawn by Fig. 6(c), where the transmission and reflection efficiencies denoted by *r*_∞_ and *r*_cx_ represent the amplitude efficiencies of the co-polarized and cross-polarized components of the reflected beam respectively. They can be analytically obtained with the help of the method of transmission matrix. To this end, note that the transmission matrix of *M*_D1_, *M*_G_ and *M*_D2_ read as

10 $${T}_{{\rm{D1}}}=(\begin{array}{cc}\frac{1}{1-r} & \frac{r}{-1+r}\\ \frac{r}{-1+r} & \frac{1}{1-r}\end{array}),$$

11 $${T}_{{\rm{G}}}=(\begin{array}{cc}{\bar{r}}_{con}-\frac{{\bar{r}}_{unc}^{2}}{{\bar{r}}_{con}} & \frac{{\bar{r}}_{unc}}{{\bar{r}}_{con}}\\ -\frac{{\bar{r}}_{unc}}{{\bar{r}}_{con}} & \frac{1}{{\bar{r}}_{con}}\end{array})$$

and

12 $${T}_{{\rm{D2}}}=(\begin{array}{cc}\frac{1}{1+r} & \frac{r}{1+r}\\ \frac{r}{1+r} & \frac{1}{1+r}\end{array})$$

respectively, where r denotes the reflection efficiency of the air-dielectric interface for the case of air side incidence, and ${\bar{r}}_{{\rm{cx}}}$ and ${\bar{r}}_{{\rm{co}}}$ are given by Eqs (1) and (2) respectively. Moreover, the transmission matrix that connects two consecutive virtual mirrors is given by

13 $${T}_{{\rm{S}}}=(\begin{array}{cc}{e}^{i(\pi /2+{\rm{\Delta }}\alpha )} & 0\\ 0 & {e}^{-i(\pi /2+{\rm{\Delta }}\alpha )}\end{array}).$$

By cascading these transmission matrixes, the reflection efficiency, i.e. *r*_∞_, is calculated. The result reads as

14 $${r}_{{\rm{co}}}\approx \frac{{r}^{2}+6r+1}{{r}^{2}-1}(i{\rm{\Delta }}\alpha +\frac{3{r}^{2}+2r+3}{{r}^{2}-1}{\rm{\Delta }}{\alpha }^{2}).$$

The above reflection efficiency can be cancelled out at the level of second order accuracy when $r=-3+2\sqrt{2}$. It turns out that the corresponding permittivity of the dielectric medium is exactly 2.

To verify the theoretical conclusion drawn by the above signal-flow chart analysis and also provide more insights, we next reexamine the proposed polarization converter with the help of the eigen-mode method. In essence, the eigen-mode method investigates the system’s responses under the situation that the system is excited using its eigen-modes. Relating to our case, the proposed polarization converter has two eigen-modes. The first eigen-mode has a polarization parallel to the grating wire and is denoted as the TE mode, while the second eigen-mode has a polarization perpendicular to the grating wire and is denoted as the TM mode.

**Figure 7:**
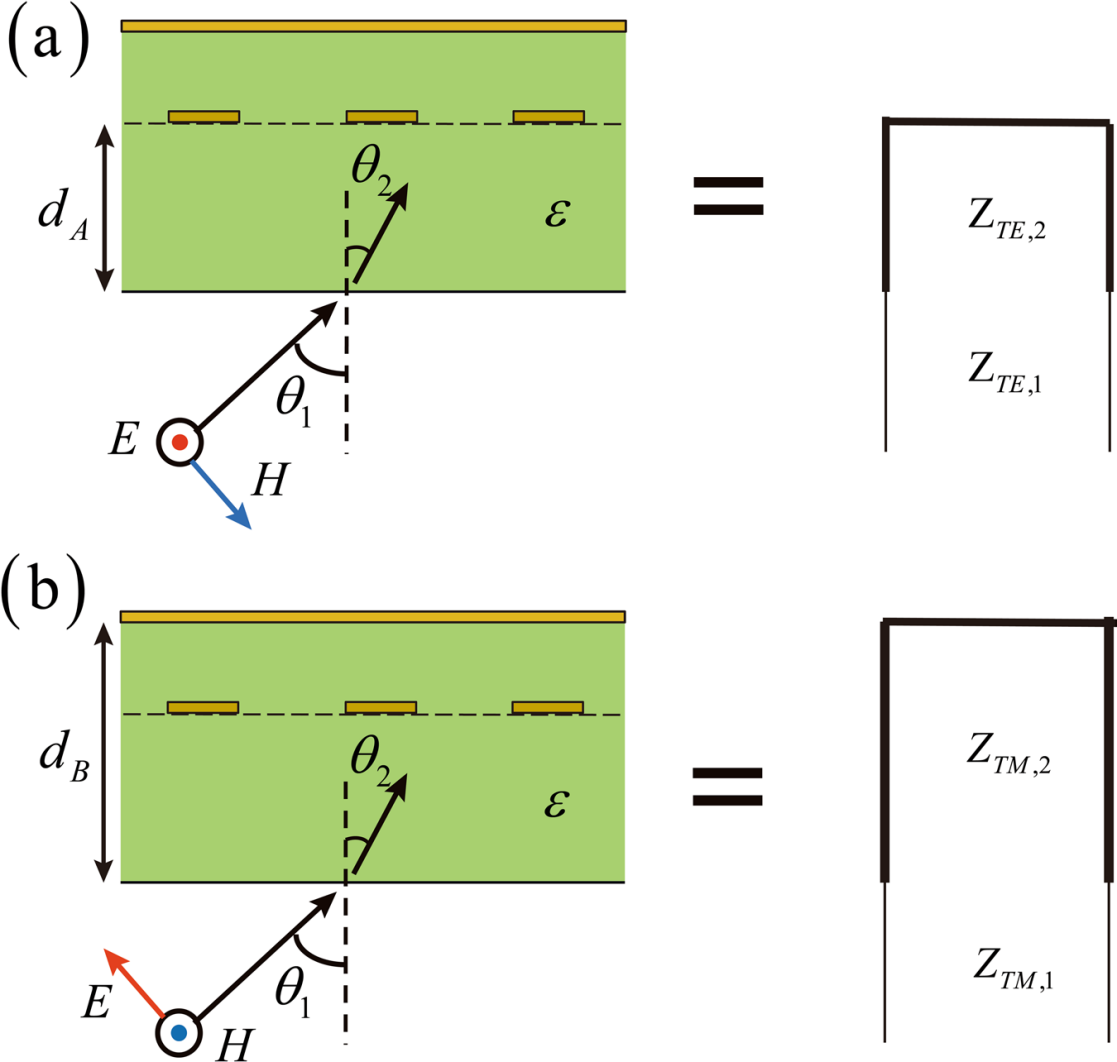
Eigen-mode picture for the general analysis of the proposed broadband half-wave plate. (**a**) Case of TE eigen-mode excitation. (**b**) Case of TM eigen-mode excitation. Note that for genericity the grating wire is positioned away from the middle plane of the dielectric slab and the incident angle is oblique.

Figure 7 illustrates the eigen-mode picture of the investigated device. For genericity, here we assume that the metal grating is placed asymmetrically inside the dielectric slab and the input wave is obliquely incident onto the device. Because the grating structure is reflective for the TE mode but transparent for the TM mode, the eigen-mode problems of the investigated device then can be analyzed using two separate transmission line models. The first transmission line model is depicted by the right part of Fig. 7(a). It is in correspondence to the case of TE mode excitation. In this situation, the oblique propagations of the TE wave in air and dielectric are modeled by two transmission lines with characteristic impedances of ${Z}_{\mathrm{TE},1}={Z}_{{\rm{0}}}\,/\,\cos \,{\theta }_{1}$ and ${Z}_{\mathrm{TE},2}={Z}_{{\rm{0}}}/(\cos \,{\theta }_{2}\sqrt{\varepsilon })$ respectively, where *Z*_0_ denotes the vacuum impedance, *θ*_1_ denotes the incident angle, *θ*_2_ denotes the refraction angle and *ε* denotes the relative permittivity of the dielectric medium. Note that the effective length of the dielectric side transmission line is ${d}_{{\rm{A}}}\,\cos \,{\theta }_{2}$, where *d*_A_ denotes the separation between the grating and the air-dielectric interface. On the other hand, the second transmission line model is depicted by the right part of Fig. 7(b). It is in correspondence to the case of TM mode excitation. In this situation, the oblique propagations of the TM wave in air and dielectric are modeled by two transmission lines with characteristic impedances of ${Z}_{\mathrm{TM},1}={Z}_{{\rm{0}}}\,\cos \,{\theta }_{1}$ and ${Z}_{\mathrm{TM},2}={Z}_{{\rm{0}}}\,\cos \,{\theta }_{2}/\sqrt{\varepsilon }$ respectively. Note that the effective length of the dielectric side transmission line is ${d}_{{\rm{B}}}\,\cos \,{\theta }_{2}$, where *d*_B_ denotes the separation between the grounded plate and the air-dielectric interface.

With the help of the two transmission line models, the reflection efficiencies for cases of TE and TM mode excitations read as

15 $${{\rm{\Gamma }}}_{{\rm{TE}}}(k)=\frac{i\,\tan (k\,\cos \,{\theta }_{2}{d}_{{\rm{A}}})-\sqrt{\varepsilon }\frac{\cos \,{\theta }_{2}}{\cos \,{\theta }_{1}}}{i\,\tan (k\,\cos \,{\theta }_{2}{d}_{{\rm{A}}})+\sqrt{\varepsilon }\frac{\cos \,{\theta }_{2}}{\cos \,{\theta }_{1}}}$$

and

16 $${{\rm{\Gamma }}}_{{\rm{TM}}}(k)=\frac{i\,\tan (k\,\cos \,{\theta }_{2}{d}_{{\rm{B}}})-\sqrt{\varepsilon }\frac{\cos \,{\theta }_{1}}{\cos \,{\theta }_{2}}}{i\,\tan (k\,\cos \,{\theta }_{2}{d}_{{\rm{B}}})+\sqrt{\varepsilon }\frac{\cos \,{\theta }_{1}}{\cos \,{\theta }_{2}}},$$

where *k* denotes the wave vector inside the dielectric medium. Using ${{\rm{\Gamma }}}_{{\rm{TE}}}$ and ${{\rm{\Gamma }}}_{{\rm{TM}}}$, a parameter *η* is then defined as $\eta (k)={{\rm{\Gamma }}}_{{\rm{TE}}}(k)/{{\rm{\Gamma }}}_{{\rm{TM}}}(k)$. Note that *η* can be used as a quantitative measure to access the polarization conversion effect of the investigated device. Specifically, *η* = −1 indicates that the investigated device is a half-wave plate; and by removing the frequency dependence of *η*, the bandwidth of the half-wave plate will be broadened.

To analytically find the broadband condition of the proposed half-wave plate, we rewrite the frequency dependent terms of Eqs (15) and (16) as

17 $$k\,\cos \,{\theta }_{2}{d}_{{\rm{A}}}={\phi }_{{\rm{A}}}(1+x)$$

and

18 $$k\,\cos \,{\theta }_{2}{d}_{{\rm{B}}}={\phi }_{{\rm{B}}}(1+x),$$

where ${\phi }_{{\rm{A}}}={k}_{0}\,\cos \,{\theta }_{2}{d}_{{\rm{A}}}$, ${\phi }_{{\rm{B}}}={k}_{0}\,\cos \,{\theta }_{2}{d}_{{\rm{B}}}$ and $x={\rm{\Delta }}k/{k}_{0}$. Note that in writing these definitions, *k*_0_ denotes the wave-vector of the central working frequency and *x* denotes the normalized frequency deviation away from the central working frequency. Moreover, since by definition ${d}_{{\rm{A}}} < {d}_{{\rm{B}}}$, we have ${\phi }_{{\rm{A}}} < {\phi }_{{\rm{B}}}$. The broadband condition of the half-wave plate can be satisfied if $\eta ({k}_{0})=-1$, $\eta ^{\prime} ({k}_{0})=0$ and $\eta ^{\prime\prime} ({k}_{0})=0$, where the primes denote the derivatives. With some manipulations it follows that these requirements are respectively met when

19 $$\varepsilon =-\tan \,{\phi }_{{\rm{A}}}\,\tan \,{\phi }_{{\rm{B}}},$$

20 $${\phi }_{{\rm{B}}}\,\sin \,2{\phi }_{{\rm{A}}}+{\phi }_{{\rm{A}}}\,\sin \,2{\phi }_{{\rm{B}}}=0.$$

and

21 $$\cos \,2{\phi }_{{\rm{A}}}+\,\cos \,2{\phi }_{{\rm{B}}}=0.$$

The coupled equations presented above allow ${\phi }_{{\rm{A}}}$, ${\phi }_{{\rm{B}}}$ and *ε* to be determined, and hence the complete set of the design parameters of the proposed half-wave plate. For simplicity, the solution searching range for ${\phi }_{{\rm{A}}}$, ${\phi }_{{\rm{B}}}$ is restricted to be within [0, π], i.e. the thickness of the dielectric slab is no more than half of the wavelength inside the dielectric medium. Because ${\phi }_{{\rm{A}}}$ is less than ${\phi }_{{\rm{B}}}$ (by definition) and the product of $\tan \,{\phi }_{{\rm{A}}}$ and $\tan \,{\phi }_{{\rm{B}}}$ must be negative [see Eq. (19)], we conclude that $0 < {\phi }_{{\rm{A}}} < \pi /2$ and $\pi /2 < {\phi }_{{\rm{B}}} < \pi$. With the help of these relations and Eq. (21), we obtain that ${\phi }_{{\rm{B}}}={\phi }_{{\rm{A}}}+\pi /2$. Plugging this result into Eq. (20), we obtain that $({\phi }_{{\rm{B}}}-{\phi }_{{\rm{A}}})\,\sin \,2{\phi }_{{\rm{A}}}=0$. Since ${\phi }_{{\rm{B}}}-{\phi }_{{\rm{A}}}\ne 0$, we obtain that ${\phi }_{{\rm{A}}}=\pi /2$ and ${\phi }_{{\rm{B}}}=\pi$. It follows that ${d}_{{\rm{B}}}=2{d}_{{\rm{A}}}$, i.e. the metal grating must be placed at the middle plane of the dielectric slab. To determine the permittivity of the dielectric slab, we linearize *φ*_A_ and *φ*_B_ as ${\phi }_{{\rm{A}}}=\pi /2+{\rm{\Delta }}{\phi }_{{\rm{A}}}$ and ${\phi }_{{\rm{B}}}=\pi +{\rm{\Delta }}{\phi }_{{\rm{B}}}$ respectively. Plugging these results into Eqs (20) and (21) and requiring the first order linearized term to disappear, we obtain that ${\rm{\Delta }}{\phi }_{{\rm{B}}}=2{\rm{\Delta }}{\phi }_{{\rm{A}}}$. Plugging ${\phi }_{{\rm{A}}}=\pi /2+{\rm{\Delta }}{\phi }_{{\rm{A}}}$ and ${\phi }_{{\rm{B}}}=\pi +2{\rm{\Delta }}{\phi }_{{\rm{A}}}$ into Eq. (19) and evaluating the limiting case with ${\rm{\Delta }}{\phi }_{{\rm{A}}}\to 0$, we obtain that *ε* = 2, which is in agreement with the theoretical conclusion drawn previously. It should be emphasized that since here in our eigen-mode based analysis, the oblique incidence condition has been assumed at the very beginning, the deduced broadband operating conditions, i.e. ${d}_{{\rm{B}}}=2{d}_{{\rm{A}}}$ and *ε* = 2, should also hold for cases of oblique incidences with arbitrary incident angles. The only difference between cases of normal and oblique incidences is the shift of the central working frequencies. Note that from ${\phi }_{{\rm{B}}}=\pi$ and $\sin \,{\theta }_{1}=\sqrt{\varepsilon }\,\sin \,{\theta }_{2}$, we find that

22 $${{\omega }_{0}|}_{{\theta }_{1}}={{\omega }_{0}|}_{0}/\sqrt{1-\frac{{\sin }^{2}\,{\theta }_{1}}{2}},$$

where ${{\omega }_{0}|}_{0}$ and ${{\omega }_{0}|}_{{\theta }_{1}}$ denote the central working frequencies for cases of normal and oblique incidences respectively. The theoretical predictions of the performance immunity to the incident angles and the shift of the central working frequency have been confirmed with the simulation results shown by Fig. 4.

The numerical simulations are carried out using the frequency domain solver of a commercially available software, i.e. CST microwave studio. In our simulations, the copper structure is treated as lossy metal while the F4BK substrate is treated as normal. The experimental measurements are performed in a quasi-anechoic chamber. A pair of linearly polarized broadband horn antennas are placed in front of a microwave absorbing screen, acting as the transmitter and receiver of the measurement system. Fabricated device samples are mounted on the microwave absorbing screen. A vector network analyzer (Agilent N5244) is utilized to measure the polarization related reflectance of the fabricated devices. The measured reflection data is normalized to the case of a bare metal plate, whose dimensions are identical to those of the fabricated sample. The discrepancies between the measured and simulated results are believed to be caused by the inaccurate dimensions of the fabricated sample and the inaccurate orientation angles of the sample and antenna placements.
